# Prescribing Personalized Exercise Programs Using Smartphone Sensors For Remote Fitness Assessment

**DOI:** 10.1093/geroni/igab046.3256

**Published:** 2021-12-17

**Authors:** Jeremy Jacobs, Ziv Yekutiel, Mical Arnon, Esther Argov, Keren Tchelet Karlinsky, Eti Benmoha, yael Netz

**Affiliations:** 1 Hebrew University of Jerusalem, Jerusalem, Yerushalayim, Israel; 2 Montfort, Montfort Brain Monitor, HaMerkaz, Israel; 3 Academic College at Wingate, Academic College at Wingate, HaMerkaz, Israel; 4 Academic College at Wingate, Wingate Academic College, HaMerkaz, Israel; 5 Montfort Brain Monitor, Montfort Brain Monitor, HaMerkaz, Israel; 6 Wingate Academic College, Wingate Academic College, HaMerkaz, Israel

## Abstract

Guidelines for physical activity emphasize multiple fitness components among people aged >65. The age-related increase in variability of fitness components necessitates accurate individualized assessment prior to optimal prescription for personalized exercise program. Accordingly, we tested feasibility and effectiveness of a novel tool designed to remotely assess balance, flexibility, and strength using smartphone sensors (accelerometer/gyroscope), and subsequently remotely deliver personalized exercise programs via smartphone. This pilot study enrolled 52 healthy volunteers (34 females) aged 65+, with normal cognition and low fall-risk. Baseline preliminary data from smartphone fitness assessment were analyzed to generate 42 fitness digital-markers, used to generate personalized exercise programs (5 times/week for 6 weeks). Programs included graded exercises for upper/lower body, flexibility, strength, and balance (dynamic, static, vestibular). Fitness was remotely assessed at baseline and after six weeks. Average age was 74.7±6.4 years; adherence was 3.6±1.7 exercise sessions/week. Significant improvement for pre/post testing was observed for 10/12 digital-markers of strength/flexibility for upper/lower body (sit-to-stand repetitions/duration; arm-lift duration; torso-rotation; arm-extension/flexion). Balance improved significantly for 6/10 measures of tandem-stance, with consistent (non-significant) trends observed across 20 balance digital-markers of tandem-walk and one leg-stance. Balance showed greatest improvement among the 37 participants exercising ≥3/week. These preliminary results serve as proof of concept among people aged >65: high adherence and improved fitness confirm the potential benefits and niche for remote fitness assessment used to generate personalized exercise programs. Future research is required to confirm the benefits among specific patient groups, such as those with frailty, deconditioning, cognitive and functional impairment.

